# Retracted: Peripheral Cytokines as a Chemical Mediator for Postconcussion Like Sickness Behaviour in Trauma and Perioperative Patients: Literature Review

**DOI:** 10.1155/2014/673464

**Published:** 2014-07-24

**Authors:** Neurology Research International

This article [1] has been retracted at the request of the author as it was submitted without the knowledge or consent of the other involved members of the research group or the author's graduate supervisor, including members deserving authorship or acknowledgement. Also, the manuscript lacked any acknowledgement of departmental and other support.
